# Supplementary material for: Impact of Dobbs v. Jackson on Abortion Access in Colorado: An Analysis of Incidence and Demographic Shifts Post-Roe
Source: Matern Child Health J. 2025 Dec 29;30(1):162–8. doi: 10.1007/s10995-025-04216-1 (PMC12992471; doi:10.1007/s10995-025-04216-1)
Supplement: Supplementary file 1 — Supplementary Material 1 [file 10995_2025_4216_MOESM1_ESM.docx]

**Supplemental tables**

Supplemental table. The mean, annual abortion incidence rounded to the nearest whole number in Colorado before (2004-2021) and after (2022-2023) Dobbs v Jackson by gestation age.

| Gestation age | Average Colorado abortion incidence before DvJ (2004-2021) | Average Colorado abortion incidence after DvJ (2022-2023) |
| --- | --- | --- |
| Less than 8 weeks | 7205 | 9927 |
| 9-24 weeks | 3112 | 4130 |
| 25 or more weeks | 14 | 235 |

Supplemental table. The mean, annual abortion incidence rounded to the nearest whole number in Texas and Oklahoma before (xxxx) and after (2022-2023) Dobbs v Jackson.

|  | Average Colorado abortion incidence before DvJ (2004-2021) | Average Colorado abortion incidence after DvJ (2022-2023) |
| --- | --- | --- |
| Oklahoma | 5200 | 0 |
| Texas | 3112 | 4130 |
